# Changes to signal peptide and the level of transforming growth factor- β1 due to T869C polymorphism of TGF β1 associated with lupus renal fibrosis

**DOI:** 10.1186/2193-1801-3-514

**Published:** 2014-09-10

**Authors:** Hani Susianti, Kusworini Handono, Basuki B Purnomo, Nashi Widodo, Atma Gunawan, Handono Kalim

**Affiliations:** Department of Clinical Pathology, Faculty of Medicine Brawijaya University/Dr. Saiful Anwar Hospital, Malang, Indonesia; Department of Urology, Faculty of Medicine Brawijaya University/Dr. Saiful Anwar Hospital, Malang, Indonesia; Department of Biology, Faculty of Science, Brawijaya University, Malang, Indonesia; Department of Internal Medicine, Faculty of Medicine Brawijaya University/Dr. Saiful Anwar Hospital, Malang, Indonesia

**Keywords:** T869C gene polymorphism of TGFβ1, Signal peptide, Urinary TGFβ1 level, Renal fibrosis, Lupus nephritis

## Abstract

Lupus Nephritis (LN) is a serious manifestation of lupus that can lead to End Stage Renal Disease (ESRD). Fibrosis is the main feature of ESRD, and it is likely influenced by Transforming Growth Factor Beta1 (TGFβ1). The T869C gene polymorphism of TGFβ1 is assumed to change the signal peptide, that has potential to interfere the urine production and renal protein expression of TGFβ1. The influence of T869C gene polymorphism on TGFβ1 production and renal fibrosis was evaluated in this study. Subjects were 45 patients LN with renal fibrosis and 45 participants without renal fibrosis as control, that were recruited from 2011 to 2013.Their urinary TGFβ1 levels and TGFβ1 gene polymorphisms were examined. All lupus patients underwent renal biopsy to assess their protein expression of TGFβ1 in the renal tissue by immunohistochemistry and their renal fibrosis by morphometry and chronicity index. Changes in the signal peptide interaction with Signal Recognition Particle (SRP) and translocon of endoplasmic reticulum were analyzed by Bioinformatics. Levels of urinary and protein expression of TGFβ1 increased in the LN with renal fibrosis group. There were significant differences in levels of urinary TGFβ1 in T, C allele and TT, TC, CC genotypes between case and control groups. Furthermore, patients with C allele are 3.86 times more at risk of renal fibrosis than T allele. The C allele encodes proline, which stabilizes the interaction of the TGFβ1 signal peptide with SRP and translocon, resulting in elevation of TGFβ1 secretion. Our results indicated that T869C gene polymorphism of TGFβ1 changes the signal peptide, that contributes to the production of urinary TGFβ1 and affects renal fibrosis in lupus nephritis.

## Introduction

One serious manifestation of lupus is lupus nephritis (LN), which occurs in 40% - 60% of patients and can lead to End Stage Renal Disease (ESRD) (Contreras et al. 2005; Mok 2005). Renal fibrosis is the principal feature of ESRD, and TGFβ1 are assumed to have a significant role in the profibrotic effect (Chatziantoniou and Dussaule 2005; Liu 2006). Regulation of TGFβ1 production is complicated, but 54% of its is thought to be mediated by genetics (Awad et al. 1998; Zuccardi et al. 2007). The T869C polymorphism is probably associated with the production of TGFβ1 (Wang et al. 2010). This polymorphism causes leucine to change into proline at codon 10 in the signal peptide of TGFβ1. The changing of the signal peptide is likely to interfere the transport of TGFβ1 to the endoplasmic reticulum that may affect TGFβ1 production and renal fibrosis. Therefore, the effects of T869C gene polymorphism on TGFβ1 transport, TGFβ1 production and renal fibrosis still need to be elucidated.

The study of TGFβ1 gene polymorphism in lupus patients in Taiwan and Japan showed that the TGFβ1 gene polymorphism was not associated with LN (Lu et al. 2004; Wang et al. 2007). However, a study in Colombia suggested that TGFβ1 gene polymorphism is associated with the occurrence of LN (Zuccardi et al. 2007). The study by Wang et al. (2005) and Hanafy and Abdo (2011) showed that T869C and G915C polymorphisms of TGFB1 gene were related to the progression of liver fibrosis in patients with chronic Hepatitis. But, study by Xaubet et al. (2003) showed that T869C polymorphism of TGFB1 gene was not related to the progression of lung fibrosis. The effect of TGFβ1 gene polymorphism in LN patients is still very little studied, especial studies that are associated with renal fibrosis of LN. The increasing expression levels of TGFβ1 in the kidneys and urine of patients with crescentic nephritis was associated with fibrosis (Goumenos et al. 2005). Otherwise, the up regulation of urinary TGFβ1 was not significantly correlated to renal fibrosis but associated with the LN activity (Chan et al. 2004). The role of TGFβ1 in LN and renal fibrosis is still debatable. Therefore, this study evaluated the correlation between the protein expression of TGFβ1 in renal tissue and urine of LN patients with renal fibrosis. Additionally, this study also examined the T869C polymorphism change interaction among TGFβ1 signal peptide with the Signal Recognition Particle (SRP) and translocon that possibly interfere with TGFβ1 transport into the endoplasmic reticulum. This phenomenon explains the role of TGFβ1 in LN pathogenesis with excessive fibrosis.

## Results

The data show that the majority of LN patients is female (Table 1), which is consistent with the theory that women are more often affected by SLE, due to the sex hormone influence (Hahn 2008). Prolonged illness in the case group was 19.88 ± 23.10 months, whereas the control group was 15.53 ± 11.89 months (p = 0.793). Levels of urinary TGFβ1 in the case group were higher compared to those of the control group. The most of case group had CC genotype (40%), whereas in the control group had TT genotype (68.8%).

**Table 1:** **Demographic and laboratory data characteristics of groups**

	Case group	Control group
	(n = 45)	(n = 45)
Gender:		
Female, *n* (%)	42 (93.3)	42 (93.3)
Male, *n* (%)	3 (6.7)	3 (6.7)
Age (years)	28.0 ± 8.3	30.1 ± 5.6
Serum Creatinine (mg/dl)	1.69 ± 3.91	0.60 ± 0.11*
Protein Urine (mg/dl)	768.12 ± 700.98	57.42 ± 56.71*
Creatinine Urine (mg/dl)	77.46 ± 59.78	135.03 ± 104.05*
TGF β1 Urine (pg/ml)	65.91 ± 91.20	23.68 ± 9.87*
Genotype T869C:		
TT, *n* (%)	12 (26.6)	31 (68.8)*
TC, *n* (%)	15 (33.4)	7 (15.6)
CC, *n* (%)	18 (40.0)	7 (15.6)*

Note: Values present as mean ± standard deviations, except gender and genotype T869C; %: percentage. * = p - value of <0.05: there is significant difference between case and control groups.

**Figure 1:**
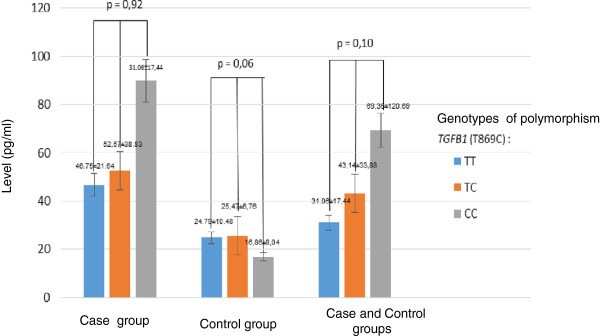
**The levels of urinary TGFβ1 in TT, TC, CC genotypes in the case and control groups were significantly different.** The CC genotype in the case group had the highest of urinary TGFβ1.

Between groups the levels of urinary TGFβ1 in the T and C allele or the TT, TC and CC genotype were significantly different (Table 2), and the highest in genotype CC (Figure 1). The data suggested that the C allele contributed to increasing the levels of urinary TGFβ1. Risk Estimation results indicate that LN patients with C allele had 3.8 times (95% CI 2.04-7.29; p = 0.00) higher incidences of fibrosis compared to T allele.

**Table 2:** **Relationship between allele and genotypes of T869C and protein expression of TGFβ1 or urinary TGF-β1 levels**

		Urinary TGFβ1 levels (pg/ml)			Protein expression of TGFβ1 in renal tissues (%)		
		Case group	Control group	p value	Case group	Control group	p value
		(n = 45)	(n = 45)		(n = 35)	(n = 10)	
Allele	T	48.99 ± 28.89	24.87 ± 9.9	< 0.01	52.68 ± 21.13	49.69 ± 26.69	<0.01
	C	78.83 ± 116.66	19.99 ± 8.37	<0.01	56.51 ± 22.42	23.57 ± 6.72	<0.01
Genotype	TT	46.75 ± 21.64	24.79 ± 10.48	0.03	47.00 ± 21.02	17.60 ± 7.12	0.14
	TC	52.57 ± 38.83	25.47 ± 6.76	0.01	63.20 ± 14.68	26.0 ± 14.71	0.01
	CC	89.78 ± 137.71	16.86 ± 8.04	<0.01	54.60 ± 25.01	27.00 ± 7.07	0.15

Note: Values present as mean ± standard deviations, %: percentage, p-value of <0.05: significant difference.

Low chronicity index scores (<4) were found in 27 (46.6%) cases and high chronicity index scores (≥4) were found in 18 cases (31.0%). Low percentage of renal fibrosis (≤5%) were found in 16 cases (30.7%) and high percentage of renal fibrosis (>5%) were found in 23 cases (44.3%) (Table 3). Further analysis showed that TGFβ1 levels had a positive correlation with chronicity index scores (r = 0.606; p <0.05), renal fibrosis (r = 0.602; p <0.05) and protein expression of TGFβ1 in renal tissue (r =0.660; p <0.05).

**Figure 2:**
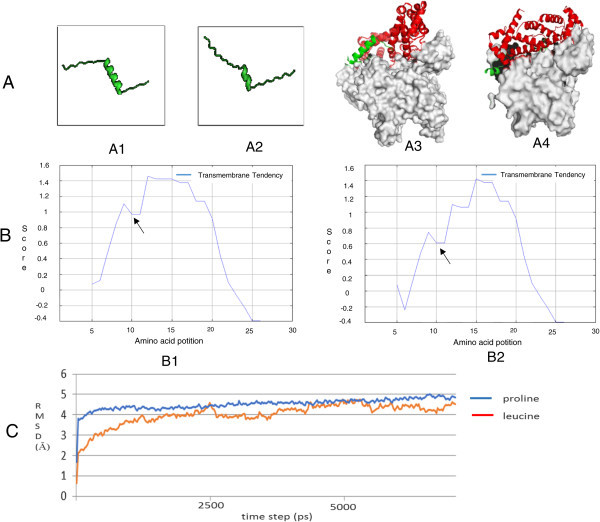
**The T869C polymorphism changes the structure and properties of the TGFβ1 signal peptide.** The polymorphism changes Leucine **(A1)** to Proline **(A2)** that breaks the helix structure that alters the interaction of TGFβ1 signal peptides (green) with Signal Recognition Particle (SRP; red) and translocon (gray) **(A3 and A4)**. The proline type of TGFβ1 signal peptide reduces the values on transmembrane tendency **(B2)** and bind more stably to the protein partner **(C)**.

The T869C polymorphism alters amino acid leucine into proline in the signal peptide of TGFβ1. The amino acid substitution affected the structure of the TGFβ1 signal peptide that in turn altered the properties of the peptide. These structural changes lead to shifting the interaction stability to the Signal Recognition Particle (SRP) and translocon and the proline effect was to reduce transmembrane tendency of TGFβ1 signal peptide (Figure 2).

**Table 3:** **Relationship between chronicity index scores and renal fibrosis to levels of urinary and protein expression of TGFβ1**

	High chronicity index score	Low chronicity index score	p value	High percentage of renal fibrosis	Low percentage of renal fibrosis	p value
	(n = 18)	(n = 27)		(n = 23)	(n = 16)	
Urinary TGFβ1 levels (pg/ml)	134.31 ± 31.65	39.32 ± 32.45	0.00	49.90 ± 30.05	34.60 ± 33.64	0.00
Protein expression of TGFβ1 in renal tissues (%)	48.78 ± 24.89	46.35 ± 24.09	0.76	60.65 ± 18.66	28.42 ± 14.13	0.00

Note: Values present as mean ± standard deviations, %: percentage, p-value of <0.05: significant difference.

## Discussion

The results of this study indicated that there were significant differences between the urinary TGFβ1 levels of LN cases with renal fibrosis and those of the control group. These results are consistent with Goumenos et al. (2005) who stated that there was an increase in TGFβ1 expression in renal tissue, and its levels in urine patients with crescentic nephritis that was associated with fibrosis. TGFβ1 induces fibrosis during tissue repair process and contribute to the pathogenesis of a variety of glomerular diseases. However, the research by Chan et al. (2004) mentioned that TGF-β1 mRNA expression was not significantly correlated with renal fibrosis but significantly correlated with SLEDAI scores and histological activity index. The difference results of our study with Chan et al. (2004) may be due to the different sampling time. A long illness case subjects in our study were 19.88 ± 23.10 months, so that stands out was the chronic state of disorder, not a state of acute inflammation.

The T869C polymorphisms of TGFβ1 gene replaced leucine into proline at TGFβ1 signal peptide. The amino acid substitution from leucine into proline changed the hydrophobic region of the TGFβ1 signal peptide that shifted its binding pattern to SRP and translocon. Proline has a cyclic structure that will change the alpha helix and increase the hydrophobicity of the core of signal peptide (Gu et al. 2012; Crilly et al. 2002). These changes might affect the transport of TGFβ1 into the endoplasmic reticulum that contributes to cytokine production. This result corresponds to Dunning et al. (2003) which found that the replacement of leucine to proline at codon 10 of TGFβ1 signal peptide could potentially alter the secretion of cytokine. Moreover, the substitution from leucine into proline led to an increase of 2.8 times of TGFβ1 secretion (Dunning et al. 2003).

The study indicated that the proline at codon ten was associated with increased TGFβ1 secretion rate and that TGFβ1 levels increased in accordance with the change of T to C allele. The TT genotype had lowest levels, and CC genotype had highest levels of TGFβ1 both in cases and control groups. Moreover, CC genotype had more prevalent in renal fibrosis and risk of ESRD. These results are consistent with Fernandez & Molina (2009) research that found the TGFβ1 polymorphism correlated with a higher level of TGFβ1 protein. This polymorphism is more common in Africans than in Caucasians, and African races have a worse prognosis in LN (Contreras et al. 2005; Fernandez & Molina 2009). Moreover, the TGFβ1 gene polymorphism may be associated with clinical manifestations and prognosis of the disease (Babel et al. 2004; August et al. 2009)

## Conclusion

T869C gene polymorphism of TGFβ1 changes properties, pattern and binding stability of TGFβ1 signal peptide with SRP and translocon that contributes to the production of urinary TGFβ1 and renal fibrosis in lupus nephritis.

## Material and methods

### Study design

A case control study was conducted. The study was conducted after approval by the ethics committee of Medical faculty of Brawijaya University, Malang, Indonesia. Informed consent was obtained from all patients for being included in the study. This study was conducted between February 2011 and October 2013 in the Department of Internal Medicine and Clinical Pathology, Dr. Saiful Anwar Hospital, Malang, Indonesia.

### Population

The cases were 45 patients LN with renal fibrosis and as the controls were 13 lupus patients without renal fibrosis and 32 healthy controls. All subjects’ blood samples taken for DNA sequencing and urinary sample for TGF β1 level examination by ELISA. The diagnosis of lupus nephritis was in accordance with the ACR (American Collage of Rheumatology) criteria 1997 and renal biopsies were performed on 58 patients with lupus. Informed consent was obtained from all patients before being included in the study.

### Laboratory assays

Urinary TGFβ1 was measured by sandwich enzyme-linked immunosorbent assay (ELISA) using a specific antibody for human TGF-β1 (Quantikine, R&D, Minneapolis, USA). Limit measurements up to 1000 pg/ml with a variation coefficient of inter assay 2.1% and 7.1% intra assay. Morning urine samples were taken aseptically, put in a sterile container, then immediately centrifuged and stored at -80°C until analyzed. Creatinine and protein levels were analyzed using the kit from Roche (Roche, New York, USA) according to the protocol in the kit.

### Isolation of DNA and sequencing

DNA isolation was performed using Dneasy Blood and Tissue Kit (Qiagen) from 200 μL venous blood samples. Samples were taken from all participants and examined by PCR using primers: 5'-d CTAGGTTATTTCCGTGGG-3' (forward primer) and 5'-d CCTTGGCGTAGTAGTCG-3' (reverse primer). Genomic DNA (100 ng) in amplification with 0.5 units of Taq DNA polymerase 2,5 μM dNTPs. PCR was conditioned 95°C for five minutes, followed by 30 cycles at 95°C for 30 seconds, then 50°C for 30 sec and 72°C for 30 secs and final extension for 10 min at 72°C (Wang et al. 2010). PCR results, then sequenced using ABI Prism Sequence Detector System (Applied Biosystems).

### In silico investigation of signal peptide of T869C TGFβ1 gene polymorphisms

3D structure (Protein Modeling) of the signal peptide of T869C TGF-β1 gene polymorphisms for leucine and proline at codon 10 types are not available in the database, so the structure was predicted using Phyre2 then validated using PROCHECK. Signal Recognition Particle (SRP ) Model 54 M domain (1QB2) and translocon model (Sec61_2wwb) were taken from PDB BANK (http://www.rscb.org) then validated using PROCHECK based on Ramachandran plot analysis. Protein docking using Vega (Escher NG) to estimate the interaction of TGFβ1 leucine and proline type with SRP-M domain and translocon. Docking results were then analyzed for molecular interactions. The stability of the bond between TGFβ1, SRP and translocon were simulated using Amber 03, Yasara Molecular Dynamic Simulation and visualized using the Pymol molecular graphics system.

### Immunohistochemistry of TGFβ1 expression in renal tissues

Specimens of tissue biopsy were preserved in 10% formalin and then cut to a thickness of 4 μm. Sections were incubated with monoclonal antibody against TGFβ1 (R&D, Minneapolis, USA). Biotin-labeled second antibody was incubated with the enzyme alkaline phosphatase labeled streptavidin. All stages were performed at room temperature, followed by washing in PBS. Staining was completed after 10 min incubation in 3,3'-diaminobenzidine in Tris buffered saline. Sections were finally counterstained in hematoxylins Mayer. The results of immunohistochemical examination were assessed by calculating the percentage of stained cells with the monoclonal anti-bodies against TGFβ1 within 500 cells of renal tissue.

### Measurement of renal fibrosis by morphometry

Morphometry analysis was used to assess fibrosis levels of the renal tissue. Staining sections used Masson 's trichrome. Digital images of the sections were photographed using Olympus microscope and Olyvia software. Assessment of the fibrosis then was performed using C5S Adobe Photoshop (Adobe Systems Corporation, San Jose, CA) (Dahab et al. 2004). Assessment of the fibrotic tissue is done by avoiding capsule and arterial adventitia areas. Fibrosis percentages were obtained by dividing the blue area (fibrotic tissue) with a total area expressed as pixels. Percentage of renal fibrosis ≤ 5% describes the state of the kidneys are normal.

### Lupus nephritis chronicity index assessment

Histopathological chronicity index of lupus nephritis was determined by renal biopsy and examined by two expert Anatomical Pathologists. The final score was obtained by summing all the scores from each of the following parameters: glomerular sclerosis, crescent fibrous structure, tubular atrophy and Interstitial fibrosis. Scores obtained from the above assessment were then added up and the number range between 1–12 (Dooley 2007). High chronicity index score (≥4) showed less response to therapy, resulting in a poorer prognosis.

### Statistical analysis

Statistical Analysis used a comparison test (t-test or Mann–Whitney and one-way ANOVA or Kruskal-Wallis), Correlation Test (Pearson or Spearmann), odd's ratio and descriptions of the structure or interaction of TGF-β1 signal peptide with SRP and translocon.

### Human rights statements and informed consent

All procedures followed were in accordance with the ethical standards of the responsible committee on human experimentation (institutional and national) and with the Helsinki Declaration of 1975, as revised in 2008. Informed consent was obtained from all patients for being included in the study.

## Abbreviations

LN: Lupus nephritis
ESRD: End Stage Renal Disease
SRP: Signal Recognition Particle
ACR: American Collage of Rheumatology.
